# Bouveret Syndrome: A Surgery-Sparing Endoscopic Approach

**DOI:** 10.7759/cureus.78412

**Published:** 2025-02-03

**Authors:** Caleb M Glover, Ali Rida, Edward Cay, Gisela Ocasio, Eugene Zolotarevsky

**Affiliations:** 1 Internal Medicine, McLaren Greater Lansing, Lansing, USA; 2 Gastroenterology, McLaren Greater Lansing, Lansing, USA; 3 Gastroenterology and Hepatology, McLaren Greater Lansing, Lansing, USA; 4 Gastroenterology, Corewell Health West Michigan, Grand Rapids, USA

**Keywords:** bouveret's syndrome, cystoduodenal fistula, holmium laser lithotripsy, primary choledocholithiasis, surgical endoscopy

## Abstract

Cholelithiasis is a common condition, and complications of gallstone disease include cholecystitis, cholangitis, pancreatitis, and, rarely, gallstone ileus or Bouveret syndrome. We present a case of a 79-year-old male with multiple comorbidities who was treated using endoscopic techniques to avoid the risks associated with surgery. The approach utilized holmium laser lithotripsy and electrohydraulic lithotripsy over multiple sessions to fragment and remove the large gallstone causing a duodenal obstruction. Conservative management of a subsequent duodenal perforation led to successful recovery. This case highlights the potential for endoscopic methods to manage gallstone ileus in high-risk patients, reducing complications and potentially avoiding surgery.

## Introduction

Bouveret syndrome is a rare but serious complication of gallstone ileus, where a large gallstone causes gastric outlet obstruction by lodging in the duodenum or stomach [1]. This condition occurs when a gallstone enters the gastrointestinal tract via a cholecystoduodenal fistula, an abnormal connection between the gallbladder and duodenum [1]. First described by the French physician Léon Bouveret in 1896, this syndrome accounts for only 1-3% of gallstone-related obstructions [2,3]. It is more common in older adults, particularly women, who are more susceptible to gallstones and related complications [4].

Management depends on gallstone size and the patient's overall health. Endoscopic removal is typically the first step for patients who are poor surgical candidates [2]. However, the success of endoscopic treatment can vary, particularly in cases involving larger stones [2]. When surgical techniques fail or the patient is not a surgical candidate, endoscopic management, which may include gastrotomy or duodenotomy for stone retrieval and fistula repair, becomes necessary. Given the health and age profile of many patients, surgery can carry significant risks, necessitating careful treatment planning. Early diagnosis and intervention are crucial to prevent complications such as bowel perforation, sepsis, or long-term obstruction [5].

## Case presentation

**Figure 1:**
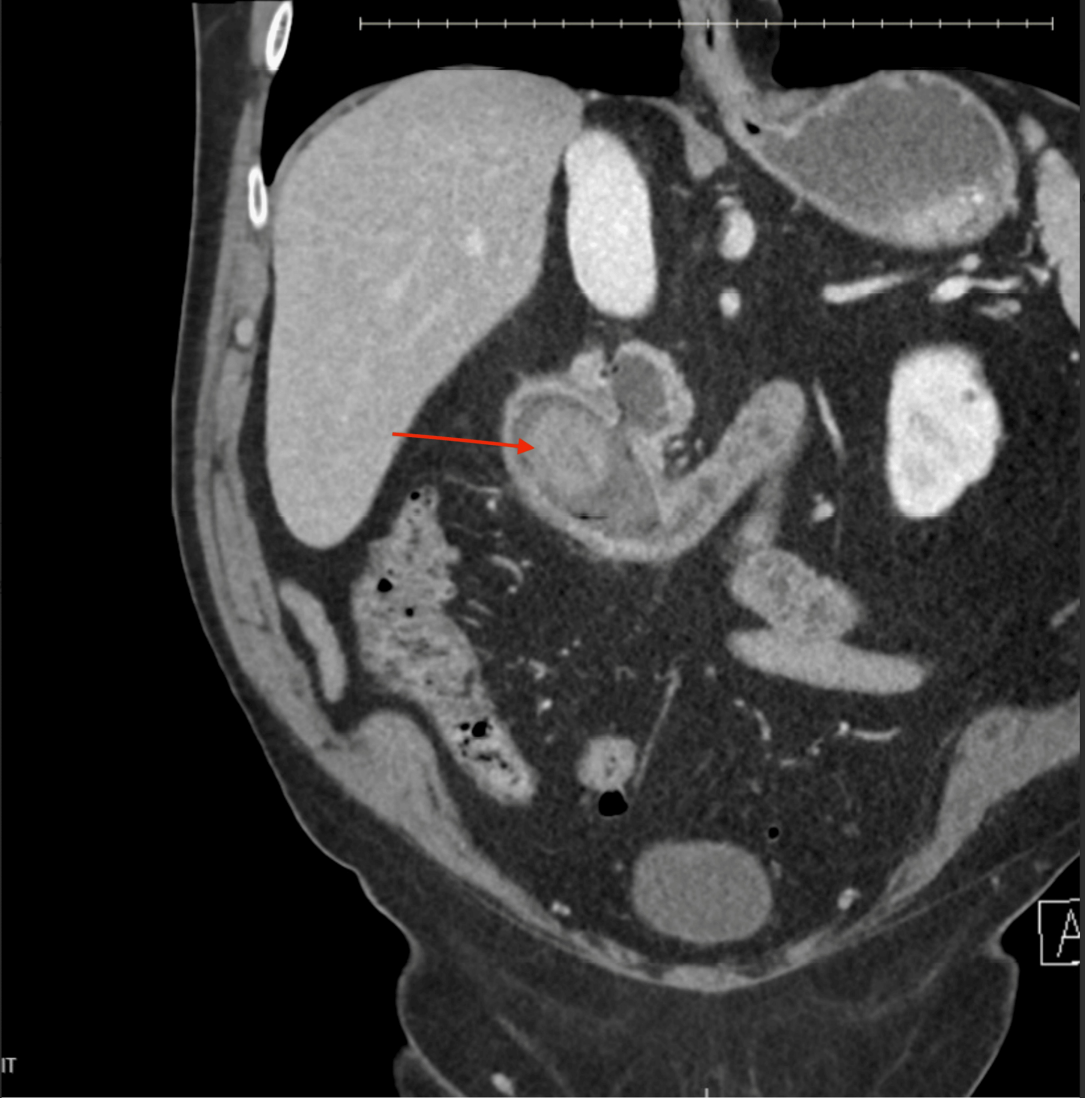
CT abdomen and pelvis demonstrating the large 5.2 x 3.2 cm gallstone (red arrow) within the duodenum along with a cholecystoduodenal fistula, consistent with Bouveret syndrome.

We present the case of a 79-year-old male with a history of obesity and gastroesophageal reflux disease who presented to the clinic with acute abdominal pain, nausea, and vomiting. An outpatient esophagogastroduodenoscopy (EGD) revealed complete duodenal obstruction with limited visualization of the causative lesion. He was referred to the emergency department, where a CT scan of the abdomen and pelvis confirmed a large gallstone, approximately 5.2 cm x 3.2 cm, causing the obstruction (Figure 1). This blockage was associated with a cholecystoduodenal fistula, consistent with Bouveret syndrome. Laboratory results demonstrated normal transaminases and mildly elevated total bilirubin of 1.4. 

**Figure 2:**
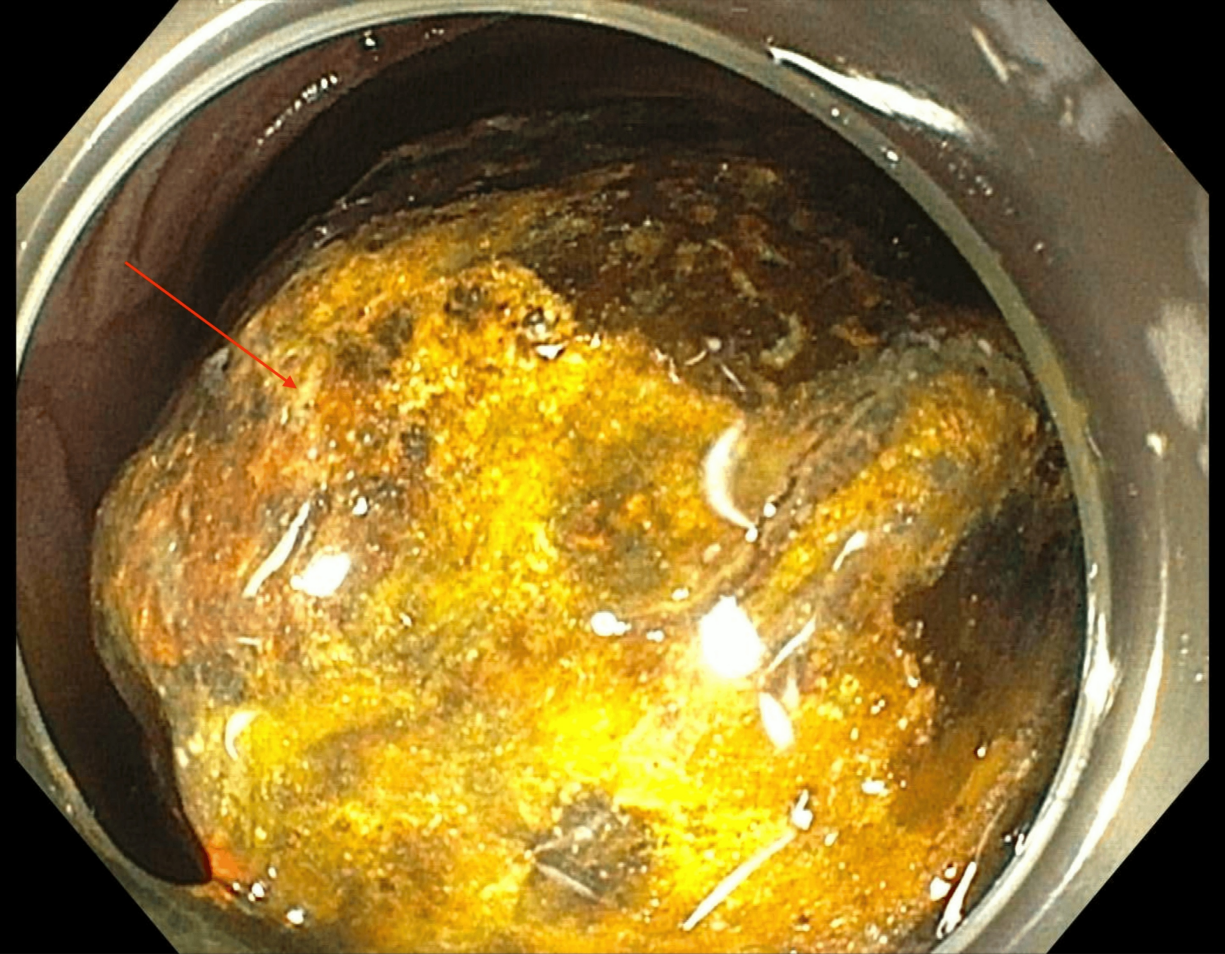
Endoscopic view depicting the gallstone (red arrow) seen during the esophagogastroduodenoscopy (EGD) prior to intervention.

**Figure 3:**
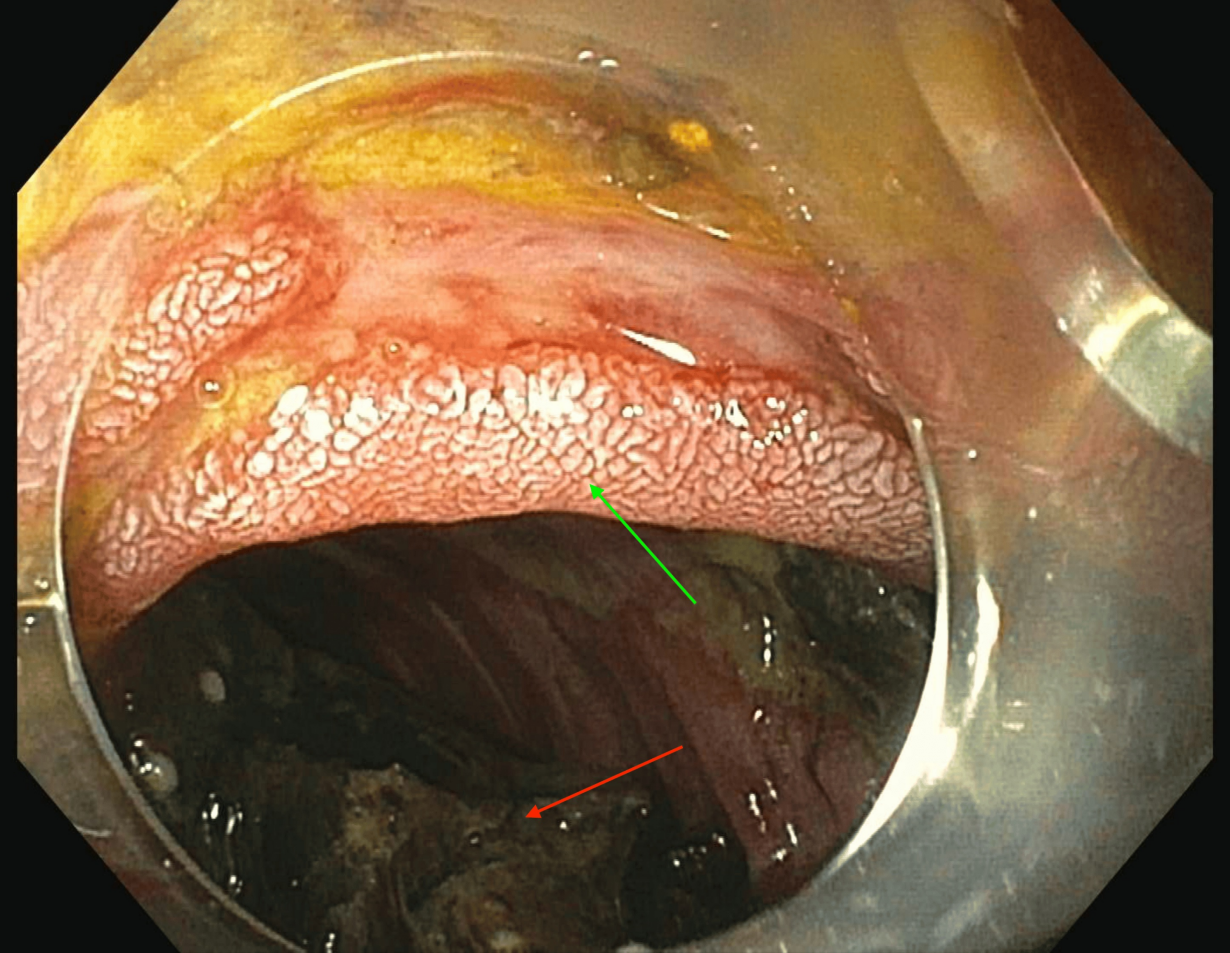
Endoscopic view of results following endoscopic intervention, showing complete resolution of the obstruction and successful removal of the gallstone. Red arrow points to gallstone debris and the green arrow points to duodenal mucosa.

Upon arrival at a tertiary care center, the patient was kept NPO (nothing by mouth) and given nasogastric tube decompression and intravenous fluid resuscitation. Given the patient’s advanced age and other health risks, a non-surgical approach was deemed appropriate. An advanced endoscopy team undertook this non-surgical approach. Over three separate endoscopic sessions, holmium laser lithotripsy was employed to fragment the large gallstone (Figure 2). The first session focused on tunneling through the stone using 40W settings and 37.9 kJ of total energy. Subsequent sessions utilized electrohydraulic lithotripsy and argon plasma coagulation to break down the remaining fragments, which were removed using a Rat tooth and Roth net. In the final session, the three remaining fragments were completely ablated using electrohydraulic lithotripsy (6,000 hits at 30/high) and argon plasma coagulation (Figure 3).

**Figure 4:**
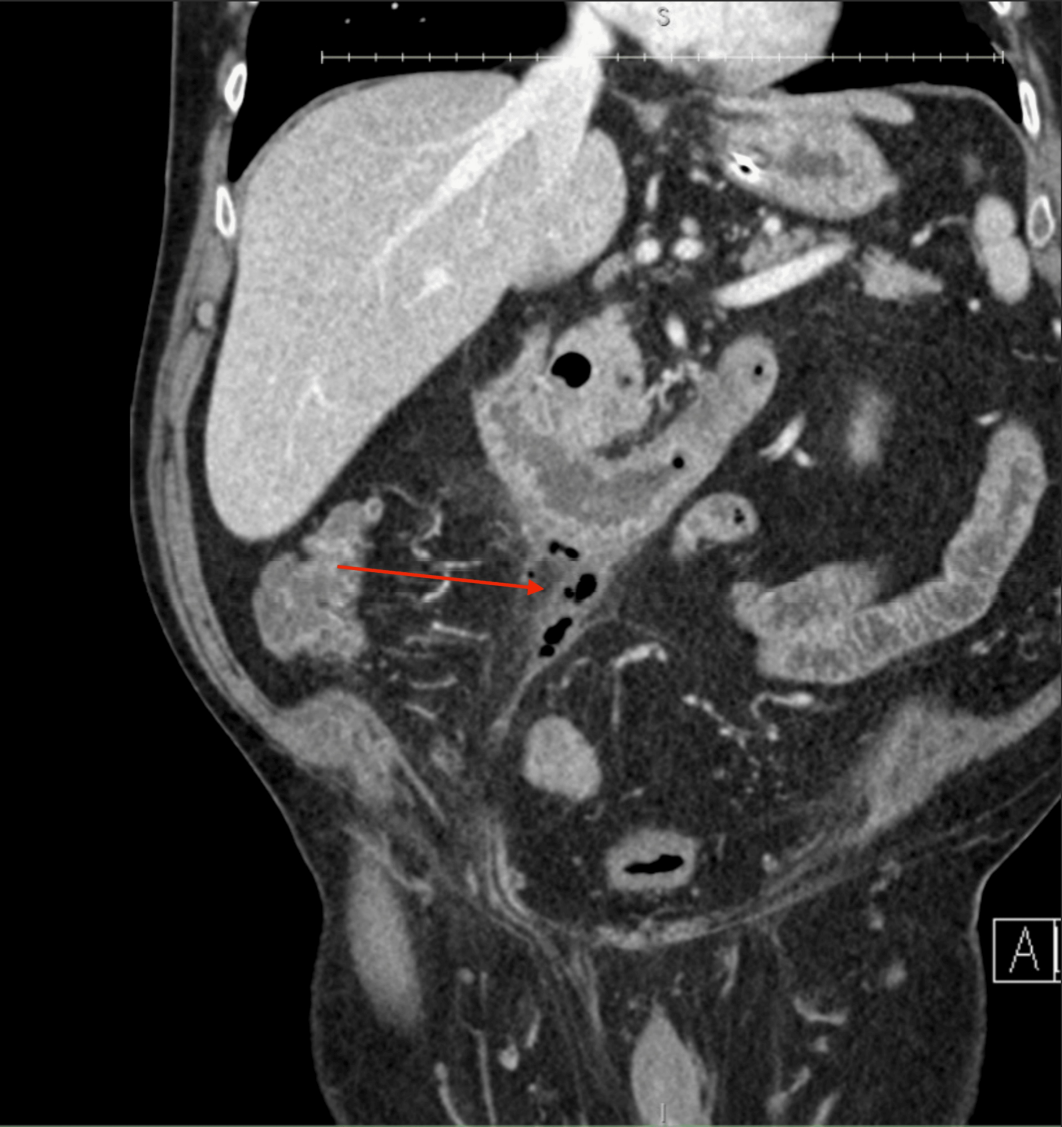
CT abdomen/pelvis demonstrating a contained perforation with hazy stranding and extraluminal gas on the anteroinferior margin of the duodenum.

Post-procedure imaging showed resolution of the obstruction but revealed a contained retroperitoneal duodenal perforation likely related to stone stasis at the D2/D3 angle, which was managed conservatively (Figure 4). A follow-up CT scan confirmed the healing of the perforation. Upon resuming bowel function, his nasogastric tube was removed, and his diet was advanced to a full liquid diet, which he tolerated well. He was discharged with instructions to maintain a liquid diet for two weeks, after which he would transition to a soft diet. At discharge, his condition was stable, with no further complications.

## Discussion

Bouveret syndrome, particularly in elderly patients with multiple comorbidities, presents significant management challenges. Surgery has traditionally been the preferred treatment for gallstone ileus, including Bouveret syndrome, typically involving gastrotomy or duodenotomy to extract the stone and often including fistula repair [6,7]. However, for elderly patients or those with significant health risks, surgical interventions pose increased complications, including infection, delayed recovery, and higher mortality rates [6]. Given the patient’s advanced age and other health concerns, a non-surgical approach was deemed more appropriate.

Advanced endoscopic techniques, such as holmium laser and electrohydraulic lithotripsy, offer a promising alternative for managing Bouveret syndrome in high-risk surgical patients [5]. In this case, a staged endoscopic approach allowed for the successful fragmentation and removal of the large gallstone, effectively avoiding surgery and minimizing associated risks. The patient’s rapid recovery, demonstrated by restored bowel function, symptom resolution, and minimal complications, underscores the effectiveness of this therapeutic strategy.

This case highlights the importance of individualized care when managing Bouveret syndrome. While surgery remains a definitive option for patients considered good surgical candidates, endoscopic intervention should be strongly considered for those at high surgical risk. The decision-making process between surgery and endoscopy must carefully weigh the risks and benefits of each approach against the potential for complications [4,8].

## Conclusions

This case demonstrates the successful management of Bouveret syndrome in a high-risk patient using advanced endoscopic techniques. While surgery has traditionally been the standard treatment for gallstone ileus, this case illustrates that endoscopy, especially when utilizing holmium laser and electrohydraulic lithotripsy, can be a safe and effective alternative for patients where surgery poses significant risks. By employing a staged endoscopic approach, the gallstone was completely removed, and the obstruction was resolved with minimal complications. Although endoscopic management carries the potential risk of perforation, these complications are often manageable conservatively, facilitating less invasive care and quicker recovery.
